# A Rare Case of Post-transcatheter Aortic Valve Implantation Complicated by Aortic Dissection and a Preoperative Anaphylactic Reaction

**DOI:** 10.7759/cureus.97077

**Published:** 2025-11-17

**Authors:** Komal Rizwan, Lenka Cagova

**Affiliations:** 1 Intensive Care Unit, Royal Papworth Hospital, Cambridge, GBR; 2 Critical Care Medicine, Royal Papworth Hospital, Cambridge, GBR

**Keywords:** antibiotics, aortic dissection, leading cardiac arrest, peri-operative anaphylaxis, prophylactic antibiotics, transcatheter aortic valve implantation (tavi)

## Abstract

Transcatheter aortic valve implantation (TAVI) is an established treatment for severe aortic stenosis. Despite its minimally invasive nature, serious complications can occur, particularly in frail, elderly individuals.

An 89-year-old, frail woman with severe symptomatic aortic stenosis and multiple comorbidities underwent elective TAVI. The procedure, planned under conscious sedation, was complicated by an anaphylactic reaction to an antibiotic, requiring emergency intubation, vasopressor support, and intensive care admission. TAVI was subsequently completed successfully. Postoperatively, she developed sudden chest and back pain, new-onset atrial fibrillation with hemodynamic instability, and a reduced level of consciousness. Transesophageal echocardiography revealed a thoracic aortic dissection (AD). Given her advanced age, frailty, and poor surgical candidacy, no further intervention was pursued. Despite maximal supportive therapy, she deteriorated and passed away peacefully.

This case highlights the challenges of managing frail, elderly patients undergoing TAVI. Although the procedure offers a less invasive alternative to surgery, severe complications such as AD may occur. Individualized risk assessment, multidisciplinary evaluation, and alignment of treatment goals are essential for optimal care.

## Introduction

Transcatheter aortic valve implantation (TAVI) has become the preferred intervention for patients with severe aortic stenosis who are deemed high risk for conventional surgical valve replacement, especially in elderly and frail populations [1,2]. As the global population ages, the number of patients eligible for TAVI continues to grow, making the management of this cohort a common and evolving challenge in cardiovascular medicine. While TAVI is generally well tolerated, it is not without significant risk, particularly in patients with multiple comorbidities.

Complications associated with TAVI, such as annulus rupture and aortic injury, are relatively rare. However, when these complications do occur, they can lead to serious consequences, especially ascending aortic dissection (AD), which is one of the most fatal complications. The mechanism for the development of ascending AD is thought to be aortic injury caused by devices, and it has been reported that most cases occur during procedures [3].

TAVI presents significant challenges in its management, not only due to the technical complexity of the procedure, but also because it is primarily performed on elderly patients who often have multiple comorbidities, making perioperative care and post-procedural recovery more intricate and demanding [4].

Anaphylactic reactions to antibiotics, though uncommon, can rapidly destabilize already vulnerable patients and complicate even well-planned procedures. Anaphylaxis-related hemodynamic instability can rarely contribute to mechanical aortic injury. An AD following TAVI is a rare but catastrophic complication, especially in those with underlying vascular fragility. Studies show that the incidence of AD after TAVI is between 0.1% and 1.9% [5].

This case highlights the critical need for meticulous perioperative planning, early recognition of drug hypersensitivity, and realistic goals of care in elderly patients. It also highlights the importance of a multidisciplinary approach in optimizing outcomes and making ethically sound decisions.

## Case presentation

An 89-year-old female presented to our specialist cardiac unit with recently diagnosed severe, symptomatic aortic stenosis. She had been experiencing increasing fatigue, reduced exercise tolerance, and occasional breathlessness over several weeks, which had significantly limited her mobility.

Her past medical history included a prior transient ischemic attack, right hemicolectomy for colon carcinoma, osteoporosis, hiatus hernia, osteoarthritis, diverticulitis, and vertebral fractures following a fall. Her known allergies included penicillin (unspecified reaction), clarithromycin (gastrointestinal symptoms and pain when swallowing), and various food allergens, including eggs, dairy products, and shellfish.

Pre-procedural transthoracic echocardiogram showed preserved left ventricle (LV) systolic function (left ventricular ejection fraction, or LVEF 55%-56%), mean aortic gradient 39 mmHg, Doppler velocity index of 0.22, and estimated pulmonary artery systolic pressure (PASP) ~40 mmHg, with normal right ventricle (RV) function. Findings were consistent with severe aortic stenosis. According to the guidelines, aortic stenosis is defined as severe in the presence of a mean gradient ≥40 mmHg, aortic peak velocity ≥4 m/s, and aortic valve area (AVA) ≤1 cm² (or an indexed AVA ≤0.6 cm²/m²).

Given the patient’s age, comorbidities, and elevated surgical risk scores (STS/EuroSCORE II) and assessment, TAVI was considered the preferred treatment over surgical replacement.

On the day of the procedure, she was transferred to the Cath lab and prepared for the planned intervention. Based on the trust’s local antibiotic prophylaxis regimen, the antibiotics were planned to be administered. Monitoring was established, and she was administered intravenous gentamicin, which was well tolerated, followed by a test dose of teicoplanin. Shortly after receiving the full dose of teicoplanin, she reported a sudden sensation of feeling hot and nausea. Examination revealed facial and upper body flushing, low blood pressure, and respiratory compromise, including tachypnoea and increased work of breathing.

A clinical diagnosis of anaphylaxis was made. Based on trust guidelines for the management of perioperative anaphylaxis, she was immediately treated with adrenaline, corticosteroids, and an antihistamine. An arterial line and central venous access were established for close monitoring and fluid administration. Her condition continued to deteriorate, with a fluctuating Glasgow Coma Scale (GCS) and worsening breathing. Owing to her condition, a decision was made to intubate her for airway protection and hemodynamic stability.

After multidisciplinary discussion and considering the urgent cardiac issue, the decision was made to proceed with the procedure itself, which was technically successful and without immediate procedural complications. The access was right radial artery (RRA) (6F), left femoral vein (LFV) (8F), and right femoral artery (RFA) (6F) - preclosed with 2× ProStyle (Abbott Laboratories, Abbott Park, IL, USA) - upsized to 10F. Over an Amplatz stiff wire (Cook Ltd., Altrincham, UK), a 14F Sentrant™ sheath (Medtronic, Minneapolis, MN, USA) was advanced. The valve was crossed with a straight-tip wire using an AL1 catheter. This was exchanged to a long wire, and then a pigtail was advanced into the LV. Circulo wire (Abbott Laboratories, Abbott Park, IL, USA) was then deployed into the LV over the pigtail catheter. The patient was paced with temporary pacing wires. The aortic valve was predilated with a 20 mm Cristal balloon (BALT SAS, Montmorency, France) under pacing at a rate of 180/min. A 26 mm Evolut FX valve (Medtronic, Minneapolis, MN, USA) was advanced but could not cross into the aorta; it was decided to place a 14F e-sheath and advance the Evolut across, then deploy under pacing at 120/min. The valve was deployed at a good height, and there was no paravalvular leak on the aortogram. The RRA was closed by a transradial (TR) band, LFV (8F) sheath was stitched in place with transpacing wires, and RFA was preclosed with 2× ProStyle. Heparin given at the initial stages was reversed with protamine.

A post-procedural transthoracic echocardiogram was performed, which showed a well-seated TAVI valve in the aortic position. Trivial to mild paravalvular leak was noted, originating from around the 2 o’clock position. LV systolic function was visually similar to baseline. No obvious pericardial effusion was seen.

The patient was transferred to the intensive care unit for recovery and was extubated two to three hours later. Initially, she remained hemodynamically stable and alert. However, later in the evening, she reported sudden-onset chest pain followed by severe back pain. She was initially hypertensive with fast atrial fibrillation, confirmed with cardiac monitoring and ECG, but shortly afterward became hypotensive. The amiodarone loading was insufficient to control this; therefore, a cardioversion was attempted, which reverted her rhythm back to sinus. The patient continued to experience recurrent episodes of hypotension requiring vasopressor support. Shortly afterward, her GCS dropped, resulting in re-intubation. 

A bedside focused intensive care echocardiography (FICE) was unremarkable, and due to her deteriorating clinical condition, she remained too unstable to be considered for transfer for a computed tomography (CT) scan.

Shortly thereafter, the patient suffered a cardiac arrest and achieved return of spontaneous circulation (ROSC) after one cycle of cardiopulmonary resuscitation (CPR). A chest X-ray was performed, which showed a left-sided massive hemothorax, and a transesophageal echocardiogram was carried out, confirming a diagnosis of AD (Video 1). The surgical team was involved and consulted, but in view of her frailty, advanced age, and comorbidities, it was decided that she would not be considered a candidate for aortic surgery. Family discussions were initiated, with multidisciplinary support for palliation.

**Video 1:** Aortic arch with aortic dissection

Despite continued organ support, her condition declined further. After discussions between the intensive care, surgical, and cardiology teams, it was determined that further life-sustaining treatments would not affect the outcome. A plan for comfort-focused care was initiated, and the palliative care team was involved to support the patient and her family. She passed away peacefully on the same night.

## Discussion

TAVI has become a widely accepted alternative to surgical aortic valve replacement in patients who are at high or prohibitive surgical risk, particularly in elderly populations. This case highlights several critical clinical challenges, including managing drug hypersensitivity in frail patients, recognizing atypical presentations of anaphylaxis, and addressing rare but catastrophic complications such as AD following TAVI. These challenges underscore the importance of multidisciplinary perioperative management and vigilant monitoring for atypical signs and potential complications to improve patient outcomes.

Mechanisms and risk factors

AD following TAVI is rare but has been documented, particularly in individuals with underlying aortic wall fragility, such as connective tissue disease or chronic hypertension. In this case, advanced age and a history of osteoporosis suggest a possible predisposition to vascular fragility. Dissection may result from mechanical stress during catheter manipulation or post-procedural hypertension, especially in a weakened aortic wall.

Anaphylaxis due to glycopeptide antibiotics such as teicoplanin is uncommon but potentially life-threatening. According to the NAP6 audit [6], among antibiotics used for surgical prophylaxis, teicoplanin allergy - although relatively rare - has been increasingly recognized. The reported incidence of anaphylaxis to teicoplanin is estimated to be around 1 in 5000 to 1 in 10,000 administrations, which is higher than previously found. The mechanism involves non-IgE-mediated mast cell activation or, less commonly, IgE-mediated hypersensitivity. Early signs may include flushing, nausea, and hypotension - features that can be mistakenly attributed to sedation or procedural stress. 

The delay in clinical deterioration after the reaction may have allowed ongoing inflammatory and hemodynamic stresses to accumulate, leading to physiological decompensation. In cases like this, the intense allergic response can cause widespread inflammation and weakening of the blood vessel walls, particularly the aorta. When combined with increased blood pressure and shear stress from physiological instability, this weakening can precipitate a tear in the aortic wall, resulting in an AD. The delayed recognition and treatment may, thus, have contributed to the progression of vascular injury and the development of this life-threatening complication.

Clinical guidelines and diagnostic approach

The 2021 ESC/EACTS guidelines [7] on valvular heart disease recommend careful pre-procedural assessment, including allergy history, frailty evaluation, and vascular access planning. In suspected anaphylaxis, early administration of adrenaline and airway protection are central to survival. Post-procedural deterioration should prompt immediate consideration of mechanical complications - pericardial tamponade, valve embolization, and vascular rupture. Transesophageal echocardiography remains a cornerstone in identifying dissection in unstable patients when transport for advanced imaging is not feasible.

Comparison with published literature

Similar cases describing dissection post-TAVI are limited but include patients presenting with late rupture or delayed chest pain without classic tearing sensations. A recent case published by Cabrita et al. [1] showed a 53-year-old female with multiple comorbidities undergoing TAVI who developed serious post-procedural aortic complications, such as AD. Another case report was published by Fujita et al. [3], describing an 82-year-old lady who underwent TAVI due to severe aortic stenosis. On the second postoperative day, she suddenly became unconscious, and a CT scan indicated an ascending AD. Unfortunately, she passed away. An autopsy revealed fragility of the ascending aorta.

This emphasizes the potential for severe, life-threatening aortic complications in high-risk patients undergoing TAVI, underscoring the importance of vigilant monitoring and prompt diagnosis.

## Conclusions

A thorough preoperative allergy assessment, with particular attention to identifying safe alternative antibiotics, is vital to minimize the risk of perioperative hypersensitivity reactions and to ensure appropriate prophylaxis. Symptoms of anaphylaxis can evolve rapidly or present atypically, and may closely mimic expected procedural side effects; such overlap can obscure the diagnosis and delay lifesaving intervention.

Clinicians must maintain a high index of suspicion and monitor patients closely. Although uncommon, delayed AD following TAVI can present as unexplained hemodynamic instability or chest pain, and should therefore be included in the differential diagnosis when postoperative deterioration occurs. In unstable patients, urgent imaging, such as CT scan and transesophageal echocardiography, provides the most accessible, rapid, and reliable bedside tools to assess for dissection, pericardial effusion, valvular complications, or other structural causes of instability, facilitating prompt management decisions.
